# The Association between Platelet Count and Acute Phase Response in Chronic Spontaneous Urticaria

**DOI:** 10.1155/2014/650913

**Published:** 2014-06-16

**Authors:** Alicja Kasperska-Zając, Alicja Grzanka, Jerzy Jarzab, Maciej Misiołek, Magdalena Wyszyńska-Chłap, Jacek Kasperski, Edyta Machura

**Affiliations:** ^1^Chair and Clinical Department of Internal Diseases, Dermatology and Allergology, Medical University of Silesia in Katowice, Ulica M. Curie-Skłodowskiej 10, 41-800 Zabrze, Poland; ^2^Chair and Clinical Department of Otolaryngology, Medical University of Silesia in Katowice, Ulica M. Curie-Skłodowskiej 10, 41-800 Zabrze, Poland; ^3^Department of Prosthetic Dentistry, Medical University of Silesia in Katowice, Plaça Akademicki 17, 41-902 Bytom, Poland; ^4^Department of Pediatrics, Medical University of Silesia, Ulica 3-go Maja 13-15, 41-800 Zabrze, Poland

## Abstract

*Background*. The platelet parameters and C-reactive protein (CRP) are markers reflecting a systemic inflammatory response. Among those, CRP is one of the major proteins helpful in determination of severity/activity of chronic spontaneous urticaria (CSU). *Aim*. To determine relationships between platelet activation indices and serum concentration of CRP, the best marker of acute phase response, and their potential clinical use in CSU patients. *Methods*. Mean platelet volume (MPV), platelet distribution width (PDW), and platelet count as well as serum CRP concentration were measured in CSU patients, showing different degrees of urticarial severity, and in the healthy subjects. *Results*. No significant differences were found in MPV and PDW between CSU group and the healthy subjects. The platelet count was significantly higher in moderate-severe CSU than that of the controls and mild CSU patients. Serum CRP concentrations were significantly higher in CSU patients as compared with the healthy subjects and significantly correlated with the platelet count in CSU patients. *Conclusions*. Acute phase response in CSU is associated with the increased number of circulating platelets in patients with more severe symptoms. It seems that simple determination of platelet size indices is not a reliable indicator of CSU severity/activity.

## 1. Introduction

Chronic spontaneous urticaria (CSU) is an inflammatory disease, characterized by acute phase response (APR) [1, 2]. In many cases it may be caused by an interactive combination of immune, genetic, and environmental factors, including infections [3–5]. In addition, the altered function of the neuroendocrine-immune system has been recognized in CSU pathogenesis [6, 7].

C-reactive protein (CRP) is a marker of systemic CSU activity, reflecting the systemic effects of inflammatory mediators, associated with the disease, including IL-6 [8, 9].

Similarly to CRP, the platelet parameters are markers reflecting the systemic inflammatory response and activity/severity of many diseases [10–12]. Moreover, such cells play an important and active role in the immune-inflammatory response [13]. Platelet volume indices, such as mean platelet volume (MPV), an indicator of platelet size, and platelet distribution width (PDW), an index of platelet size heterogeneity, have been shown to be the markers of platelet function and activation [10–12]. Higher MPV was associated with low-grade inflammatory conditions and a variety of risk factors of cardiovascular disorders [10, 14].

On the one hand, CSU is associated with an increased level of CRP and IL-6, parallel to the disease severity/activity [9, 15]. Interestingly, it is known that IL-6 stimulates the synthesis of CRP and thrombocytosis [16, 17]. Data regarding platelet activation markers in CSU are scarce and contradictory [15, 18–21]. Taken together, it seems reasonable to determine the relationships between the platelet characteristics (count, MPV, and PDW) and serum concentration of CRP, the best marker of acute phase response, as well as their potential clinical use in CSU patients.

## 2. Material and Methods

Sixty-six patients (20 men and 46 women; median age 38 years) with active chronic spontaneous urticaria of 12 months mean disease duration (range 4–52 months) were selected for the study. Physical urticaria was studied by history and/or diagnostic tests (3 patients had concomitant dermographism).

Urticaria activity score (UAS) was estimated during four days and on the day of blood sampling: (no wheals = 0, 1–10 wheals = 1, 11–50 wheals = 2, >50 wheals = 3) and pruritus intensity (no = 0, mild = 1, moderate = 2, and severe = 3). UAS scores were as follows: daily (minimum = 0; maximum = 6) and four days by adding the daily score values (minimum = 0; maximum = 24) [22]. The UAS was graded as follows: 0–8 (mild), 9–16 (moderate), and 17–24 (severe). Our study comprised 28 patients with mild and 38 patients with moderate-severe urticaria symptoms as per the four days UAS. Each patient underwent the following investigations: routine laboratory tests (full blood count, urine analysis, ESR, C-reactive protein, serum glucose, hepatic functions, and creatinine), immunoglobulins (IgG, IgA, IgM, and IgE) and complement in serum/plasma, stool (for parasites), hepatitis serology (anti-HCV, HBsAg), antinuclear and anti-thyroid microsomal antibodies, thyroid function and* H. pylori* tests, and abdominal ultrasonography.

None of the examined subjects had taken any oral corticosteroids or antidepressants within 8 weeks or antihistamines within at least 4 days before the study. Autologous serum skin test (ASST) [4] and other investigations had been performed to exclude any known causes of the diseases or the concomitant diseases. Among those, routine dental and ENT consultations were performed to exclude the infectious foci. The diseases/conditions and drugs, which are known to be associated with changes in platelet function, have been excluded, including smoking, obesity, diabetes mellitus, hypertension, coronary, peripheral arterial disease, valvular heart disease, stroke and pulmonary embolism, and autoimmune diseases. There were no differences in cholesterol levels between the groups.

The control group consisted of 36 sex-, age-, and BMI (<25) matched healthy subjects. None of the controls took any medication for at least 14 days before the study.

The Ethics Committee of the Medical University of Silesia approved the study.

## 3. Blood Collection

Fasting blood samples containing tripotassium ethylenediaminetetraacetic acid (EDTA) were obtained between 7 and 9 a.m. by antecubital puncture. Analyses were performed immediately after sampling to prevent in vitro platelet activation.

## 4. Assay of CRP

Serum C-reactive protein (CRP) concentrations were assayed using Cobas 6000 analyzer with c501 module (Roche, Switzerland).

## 5. Assay of the Platelet Count and Indices

The platelet count, mean platelet volume (MPV), and platelet distribution width (PDW) were analyzed using Sysmex XT-2000i automated hematology analyzer (Sysmex, Japan).

### 5.1. Statistical Analysis

Results are expressed as median and interquartile ranges. Because data were not distributed normally, nonparametric tests were used. Kruskal-Wallis variance analysis was used to screen differences between the groups. Mann-Whitney *U*-test was used to compare data between the patient groups and the normal subjects. Spearman's rank test was used for correlations. The probability value of *P* < .05 was assumed significant.

## 6. Results

### 6.1. Platelet Count and Indices

The comparison of platelet parameters of CSU patients and the healthy subjects is presented in Table 1.

No significant differences were found in MPV and PDW between CSU groups and the healthy subjects. There were no significant differences in platelet count between the CSU patients (as a whole) and the healthy subjects. However, platelet count was significantly higher in moderate-severe CSU than those of the controls and mild CSU patients.

### 6.2. Serum CRP Concentration

The comparison of laboratory parameters of CSU patients and the healthy subjects is presented in Table 1.

Serum CRP concentrations were significantly higher in CSU patients as compared with the healthy subjects. In addition, there were significant differences in serum CRP concentrations between patients with mild and moderate-severe CSU as well as the healthy subjects.

No significant differences in serum CRP concentrations between ASST(+) and ASST(−) CSU patients were observed.

### 6.3. Associations

The correlation in CSU patients and the controls is presented in Table 2. Serum CRP concentration significantly correlated with platelet count in CSU patients. Significant correlations were found between MPV, PDW, and platelet count in the healthy subjects. In addition, significant correlation was shown between platelet count and PDW in SCU group.

Also there were no significant correlations between the disease duration and CRP, the platelet indices (platelet count, MPV, and PDW).

## 7. Discussion

As expected, we confirmed the previous findings indicating that patients with CSU, particularly those with a more severe disease, show signs of the low-grade inflammation as supported by the elevated concentration of CRP in serum [15, 23, 24].

It has been reported that platelet count is not increased in CSU [18]. Unfortunately, the patients were not divided by the disease activity/severity. Similarly, there were no differences in platelet count in other studies [15, 19]. In the present study, like in the previous one, there was no significant difference in platelet count between CSU patients (as a whole) and the controls. However, the platelet count was elevated in moderate-severe CSU, as compared to mild CSU and the controls. Similarly, mean platelet number was significantly higher in children with CSU when compared to the healthy children [20].

In our study, patients with mild and moderate-severe CSU showed all values of the platelet count within the normal range. Interestingly, the platelet count correlated with serum CRP concentration, a marker of urticarial inflammation, which is known as parallel to the disease severity/activity, thus probably reflecting the same characteristic of the inflammatory response in CSU.

Inflammatory thrombocytosis is thought to be related to acute phase reactants, which may act through thrombopoietin (TPO) to increase the platelet count [17]. It has been hypothesized that, in inflammatory states and malignant diseases, thrombocytosis might be mediated through an IL-6–induced increase in TPO levels [16, 17]. It is well known that CSU is associated with activation of APR and increased concentration of IL-6. Our study showed significant correlation between platelet count and serum concentration of CRP, the best marker of APR stimulated by IL-6.

It is recognized that an inverse relationship occurs normally between MPV and the platelet count in healthy subjects, which was also confirmed in our control group [25]. In contrast there was no significant correlation between the two parameters in CSU patients, characterized by increased platelet count.

The similar MPV in mild and more severe CSU patients as well as in healthy subjects observed in our study is not consistent with those indicated by other studies. In contrast, the previous reports showed that MPV is increased [15, 19] or decreased [20] in CSU patients.

MPV was significantly lower in children with CSU when compared to healthy children [22].

On the other hand, it has been reported that antihistamines-resistant CSU was characterized by higher MPV and statistically significant elevation of liver transaminases, which may be related to hepatic fat accumulation [19]. However, it has been demonstrated that patients with nonalcoholic fatty liver disease had higher MPV [26].

Magen et al. collected prospective data for 373 CSU patients and 46 healthy controls, suggesting that increased MPV may be an indicator for increased disease activity/severity in CSU patients with ASST-positive response, but not in ASST-negative group [15]. In this study, anti-TPO and anti-thyroglobulin antibodies were positive in 34.6% and 19.3% of patients with positive and 21.8% and 14% negative ASST, respectively [15]. Interestingly, it has been demonstrated thatmean platelet volume (MPV) was increased and correlated only with anti-thyroid peroxidase (TPO) antibodies as well as MPV values were increased after subclinical hypothyroidic patients became euthyroid [27, 28]. In contrast, we showed that MPV and PDW values did not differ significantly between CSU patients, regardless of the disease activity/severity and ASST response as well as when compared with the healthy subjects. Our sample size was smaller than that of Magen et al. [15]. In addition, our patient had no thyroid antibodies and normal transaminases level as compared with healthy subjects. However, in the previous study the percentage of patients with thyroid autoimmunity was similar in the CSU groups with positive and negative response to ASST [15]. The reason for such discrepancy is unclear and all our attempts of explanation have been merely speculative.

The variances between the studies on platelet indices in CSU may result from different criteria used to classify the patients and/or from the technical reasons. Although results of studies evaluating MPV and PDW in various diseases point to some potential usefulness in the clinical practice, there are several limitations which depend on the number of technical criteria, including time and method of analysis, or anticoagulant used [11]. In addition, the conflicting data regarding MPV and PDW in CSU may be due to the failure to rule out confounding factors (especially in retrospectives analysis), including diabetes mellitus, hypertension, smoking, obesity, arterial diseases, and autoimmune diseases [29–34]. Currently, almost all diseases may increase or decrease MPV/PDW; therefore, it is difficult if not practically impossible to exclude all MPV-related diseases and conditions [35].

Since the conflicting data do exist, some extensive multicenter studies should be performed to resolve the controversy.

MPV and PDW, commonly used as a measure of platelet size, indicate the rate of platelet production and platelet activation. Increased MPV and PDW may indicate enhanced platelet activation in CSU, which is in contrast with previous observation regarding the systemic release of platelet factor 4 and beta-thromboglobulin, the indices of platelet activation [18], and in agreement with the study by Chandrashekar et al. [21]. Very importantly, both the prothrombotic condition and the hypercoagulable state are not the established features of CSU. It has been demonstrated that SCU is associated with activation of the extrinsic (tissue factor) pathway and fibrinolysis with no features of the intrinsic pathway activation and thrombophilia [36, 37].

The previous studies did not provide any information regarding relationship between the platelet size indices and CRP concentration in CSU patients. We found that MPV and PDW did not correlate with serum CRP concentration in CSU groups.

### 7.1. Limitations of the Analysis

The main limitation of our study was the relatively small sample size which was due to more homogeneous group of patients. Nevertheless, we were able to detect some statistically significant differences in platelet count, between CSU patients and the healthy subjects as well as to detect an activity/severity-dependent diversity of the platelet count.

The strength of our analysis was the consistent and comparable group of CSU patients and the well-matched controls.

### 7.2. Clinical Implications

MPV and PDW values were statistically similar between the healthy subjects and patients with mild and moderate-severe CSU, effecting in failure to detect patients with more severe symptoms. The platelet count was higher in moderate-severe CSU patients, although all values were found within the normal lab range. So far, apart from CRP, none of the parameters seems effective and reliable enough to distinguish more severe CSU from milder CSU throughout numerous cases. Although the significance of MPV and PDW, as an important predictor for many diseases, is increasing, it is still the subject of debate and relevant experience is scarce, especially in CSU. Comparison of platelet size indices with other potential biomarkers is needed to characterize comprehensively its potential use in monitoring the CSU patients.

### 7.3. Conclusions

MPV and PDW values of symptomatic patients with mild and moderate-severe CSU were not markedly distinct from those of the control group. Moreover, we found that MPV and PDW were not correlated with serum CRP concentration in the groups. However, we found higher platelet count in CSU patients with more severe disease activity and a positive correlation between the platelet count and serum CRP concentration. Therefore, we suggest that acute phase response in CSU is associated with the increased number of circulating platelets in patients with more severe symptoms.

It seems that simple determination of the platelet size indices is not a reliable indicator of CSU severity/activity and appears useless as a confirmatory test for CRP.

However, due to the conflicting data regarding platelet function/activation, it is needed to perform further investigations, comprising larger groups of patients in order to assess the clinical usefulness and validity of the platelet count and indices for evaluation of the disease activity/severity in CSU.

## Figures and Tables

**Table 1:** Laboratory characteristics of patients with CSU and the controls.

Parameter	Control (*n* = 36)	CSU (whole) (*n* = 66)	CSU Mild (*n* = 28)	CSU moderate/severe (*n* = 38)	*P*			
					CSU versus control	CSU mild versus control	CSU moderate/severe versus control	CSU moderate/severe versus mild
PLT (×10^9^/L)	267 (203–296)	269.5 (229–304)	236 (201–269)	289 (257–308)	0.34	0.16	0.01	0.00028
MPV (fl)	7 (8.3–7.17)	7 (6.5–8.0)	6.8 (6.5–7.35)	7.4 (6.5–8.2)	0.73	0.29	0.76	0.13
PDW (%)	16.9 (16.5–17.3)	17 (16.5–17.6)	17.0 (16.6–17.7)	17 (16.3–17.4)	0.90	0.12	0.87	0.2
CRP (mg/dL)	1.0 (0.9–1.1)	6.35 (2.4–10.2)	2.1 (1.5–2.95)	9.9 (8.4–12.9)	<0.0001	<0.0001	<0.0001	<0.0001

CRP: C-reactive protein; PLT: platelets; MPV: mean platelet volume; PDW: platelet distribution width; CSU: chronic spontaneous urticaria.

Data shown are median (interquartile range).

**Table 2:** Correlation in CSU patients and the controls.

	CSU				Control			
	CRP		PLT		CRP		PLT	
	*r*	*P*	*r*	*P*	*r*	*P*	*r*	*P*
MPV	0.08	0.51	−0.01	0.91	−0.03	0.86	−0.44∗	0.007
PDW	−0.18	0.14	−0.26	0.03	0.11	0.51	−0.38∗	0.02
PLT	0.37	0.002	—	—	−0.008	0.96	—	—

CRP: C-reactive protein; PLT: platelets; MPV: mean platelet volume; PDW: platelet distribution width; CSU: chronic spontaneous urticaria.

*r*, Spearman correlation factor.

*P < 0.05—significant correlation.
